# Evaluating the Influence of Two Different Red Wines on the Physicochemical Properties, Volatile Compound Profiles, and Sensory Attributes of Wine-Soaked Pressed Cheeses

**DOI:** 10.3390/foods14203475

**Published:** 2025-10-12

**Authors:** Paulina Freire, Daniel Olmos, Miguel A. Pedroza, Jack Adamson, Reem Elkhalil, Madison Atwood, Justin P. Miller-Schulze, Carmen C. Licon

**Affiliations:** 1 Department of Food Science and Nutrition, California State University, Fresno, CA 93740, USA; paulinaf@mail.fresnostate.edu (P.F.);; 2 Department of Wine and Viticulture, California Polytechnic State University, San Luis Obispo, CA 93407, USA; miguelp@calpoly.edu; 3 Department of Chemistry, Sacramento State University, Sacramento, CA 95819, USA; jadamson@csus.edu (J.A.); reemelkhalil@csus.edu (R.E.); maddieatwood@csus.edu (M.A.); j.miller-schulze@csus.edu (J.P.M.-S.); 4 Dairy Products Technology Center, California Polytechnic State University, San Luis Obispo, CA 93407, USA

**Keywords:** wine-soaked cheese, Cabernet Sauvignon, Alicante Bouschet, esters, ketones

## Abstract

This study evaluated the effects of wine-soaking on cow’s milk pressed cheese properties and developed a standardized cheesemaking procedure. Cheese was soaked in Cabernet Sauvignon and Alicante Bouschet red wines for two soaking periods of four days after the brining process. The physicochemical, microbiological, and volatile composition were evaluated, along with consumer sensory evaluation. After 60 days of ripening, wine-soaked cheeses had statistically lower salt and moisture levels, with higher protein and fat content than the unsoaked cheeses. Alicante Bouschet cheeses have a darker purple-red color than Cabernet Sauvignon. The microbiological analysis found no significant differences across treatments and samplings. The most representative volatile compounds in wine-soaked cheeses were esters and ketones. Principal Components Analysis on the volatile compounds showed a clear separation between the two wine-soaked cheeses and the control cheese. For example, Cabernet-soaked cheese had higher levels of phenylethyl alcohol and 2-phenylethyl acetate (floral aromas), while Alicante-soaked cheese was distinguished by nonanal (fruity and grassy aroma). Sensory results showed preferences for the overall liking, flavor, and rind color for the wine-soaked cheeses over the control. Consequently, a standardized recipe for wine-soaked pressed cheese was developed, along with specific parameters for the soaking process to ensure a well-received product.

## 1. Introduction

The availability of different cheese products is an important factor for growth in the cheese market. Artisan and ethnic cheese production has become a significant force shaping the global dairy sector, particularly in the United States [1]. In addition, cheese manufacturing consistently delivers higher returns than fluid milk [2], underscoring the transformative potential of diversifying into innovative cheese products. Consequently, specialty cheeses and artisan cheeses have become very popular on a global scale. In fact, artisan cheese production in the United States has grown significantly in the past two decades [1]. Parallel to this, the global wine industry continues to expand [3], and merging these two products into a single, signature innovation: wine-soaked cheese has shown to be promising. In fact, pairing cheese and wine is a popular culinary experience [4]. The practice of wine-soaking has been particularly preserved in Mediterranean regions [5]. Wine-soaked cheeses, such as *Murcia al Vino* (Spain) [6], or *Ubriaco* and *Occelli* (Italy) [7], belong to a category of specialty cheese that builds on the synergy between local wines and cheeses to enhance the flavor profile, preservation, and distinction of the regional cheese. Research into wine-soaked cheeses has revealed that the soaking process profoundly influences multiple aspects of cheese quality and composition [8]. For example, the wine treatment affects the physicochemical properties such as moisture content, pH, and mineral composition contributing to their unique identity [9]. In addition, López et al. [6] reported that the textural properties changed due to proteolysis patterns. Similarly, lipolytic activity can be impacted by wine immersion, leading to distinct volatile organic compound profiles that define the sensory characteristics of this particular cheese products [10].

Volatile organic compounds (VOCs) serve as the primary drivers of aroma and flavor in cheese and wine, with their composition and concentration determining the distinctive sensory profile that characterizes each style and variety [11]. Various groups of volatiles have been identified as being important for the final taste and aroma of cheese such groups are fatty acids, esters, aldehydes, alcohols, ketones, hydrocarbons, and sulfur compounds [12]. For example, Rizzo et al. [13] found the aroma-active compounds profile in cheddar cheese smoked with the burning of different woods, while Faulkner et al. [14] examined the impact of dairy cattle foraging on the volatile and sensory properties of the milk generated. The number of identified VOCs in such studies is typically over 50, as esters, alcohols, ketones, and aldehydes are all commonly detected in this analysis [11].

The regional and small-scale nature of wine-soaked cheese production has left standardized methods poorly documented, and to the best of authors’ knowledge, none of the published references have compared the use of different wines during two soaking periods [6,7,15,16]. Standardizing a methodology and understanding of the soaking conditions and wine characteristics will foster new product categories, enhance global market diversity, and strengthen cross-sector linkages between dairy and viticulture. Therefore, this study evaluated the impact of wine-soaking with two regional Central Valley wines (Cabernet Sauvignon and Alicante Bouschet) on the physicochemical, microbiological, sensory, and volatile properties of pressed cow’s milk cheese.

## 2. Materials and Methods

### 2.1. Cheesemaking

Cheeses were manufactured across two different production days (one for each wine type) where each day produced two batches: one for control and one for wine-soaked cheeses. Therefore, each wine treatment included its own matched control to isolate the soaking effect for each wine. The manufacturing was performed using 75.7 L of cow milk obtained from California State University, Fresno Dairy Unit at the Fresno State Creamery. Milk was transferred into a 113 L stainless-steel vat equipped with temperature control. Once the milk reached a temperature of 31.1 °C, 7.6 units of mesophilic (Flora Danica, Chr. Hansen, Madison, WI, USA) and thermophilic (ST-B01, Chr. Hansen, Madison, WI, USA) cultures were added. The starter cultures contained *Leuconostoc mesenteroides* subsp. *cremoris* (LMC), *Lactococcus lactis* subsp. *cremoris* (LLC), *Lactococcus lactis* subsp. *lactis* (LL), and *Lactococcus lactis* subsp. *lactis* biovar diacetylactis (LLD). After 35 min, CaCl_2_ was added (32% stock solution; 30 mL of CaCl_2_ in 30 mL deionized (DI) water) in milk and stirred frequently. Coagulation of milk was performed at 31.1 °C for 30 min after the addition of recombinant rennet chymosin (Maxiren 180, dsm-firmenich, Seclin, France) at a concentration of 5000 IMCU/15 DI water (%*v*/*v*). The curd was cut into 8–10 mm cubes using the stirring-cutter, stirred along with a gradual increase in temperature until reaching 42.8 °C and heated up for about 40 min. After draining the whey, curds were placed into cheese molds weighing approximately 0.91 kg lined with a cheesecloth. The forms were fitted with caps and pressed at 10 Pa with increases of 5 Pa every 30 min until the pH was 5.2 or below. The cheeses were turned upside down twice each pressure step during the first hour, and the cheesecloth was removed after the first pressure step. The cheese pieces were placed into a brine solution (23% NaCl, *w*/*v*) at 4 ± 1 °C for three days. After brining, the cheeses were allowed to dry for an additional three days. Following this, a batch of cheese was soaked in 5 L of wine for four days and then dried for another two days. The cheeses were re-soaked in the same wine for another four days. The cheeses were flipped every two days during the soaking process. Once the cheeses were manufactured and soaked in their respective wine treatments or not soaked for the control, they were vacuum sealed and stored for 60 days. Throughout this process, the cheeses were aged and/or dry at 12 ± 1 °C and 85% relative humidity. Commercial Cabernet Sauvignon and Alicante Bouschet wines (vintage 2022) from the Viticulture and Enology Program winery at California State University, Fresno, were used for cheese soaking. The wines had fermentations performed in stainless steel vats and aging for one year in a mix of American and French oak barrels.

### 2.2. Cheese Sample Preparation for Analyses

One full cheese piece per treatment was pulled out for sampling during the ripening process. The sampling procedure was conducted on days 3, 30, and 60 after production, where microbiological and chemical composition were carried out. In addition, color and sensory analyses were performed after the 60th day. All measurements were conducted at the Dairy Diagnostics Lab at California State University, Fresno. Cheese samples were taken with a sterilized probe and 2 cm of the cheese rind were removed prior to obtaining the samples. All the compositional, microbiological and volatile profile analyses were carried out at least in duplicate for each batch of cheese.

### 2.3. Microbiological Analysis

A cheese sample of 11 g was diluted with 99 mL of sterile buffered peptone water (Oxoid Ltd., Basingstoke, UK) in a stomacher bag and homogenized for 2 min with a Stomacher (Seward Ltd., London, UK). Dilutions series were performed with sterile buffered phosphate water and 1 mL of this dilution was transferred to 3M Petrifilm™ plates for microbiological counts. For total plate counts (TPC), 3M Petrifilm™ AC plates were incubated for 48 ± 3 h at 32 ± 1 °C [17]. For coliform counts, 3M Petrifilm™ HSCC were incubated for 24 ± 2 h at 30 ± 1 °C [18]. For yeast and mold, 3 mL of the dilution was transferred to 3M Petrifilm™ RYM plates and incubated for 72 ± 2 h at 28 ± 1 °C. Finally, Listeria was analyzed at the end of ripening, after 60 days after the cheese production, where 3 mL of the dilution was transfer to a 3M Petrifim™ Environmental Listeria plate and incubated for 28 ± 2 h at 37 ± 1 °C. All determinations were made in duplicate and expressed as log colony-forming units per gram of cheese (cfu/g).

### 2.4. Physical–Chemical Analysis

Dry matter, fat, protein, and sodium content were obtained by using a NIRSTM DS 2500 F (Foss Electric A/S/, Hillerod, Denmark). The interior of the cheese was grated to acquire uniform grain size and 40 g of cheese from each treatment was placed on a cylinder glass for measurements.

In addition, color measurements were made with a Minolta CR-400 colorimeter (Konica Minolta Sensing Inc., Osaka, Japan). The colorimeter was calibrated to white calibration plate (Number 113331100, Y = 93.1, x = 0.3160, y = 0.3323, Konica Minolta Sensing Inc., Osaka, Japan) before use. Obtained values were expressed in terms of Commission Internationale d’Éclairage (CIE) L*, a*, and b* corresponding to brightness, redness, and yellowness, respectively. The color measurement was directly measured in the transversal surface of the wine-soaked cheese samples [19]. For each treatment, cheese soaked in Cabernet Sauvignon or Alicante Bouschet, three measurements were collected from two cheese samples (3 measurements × 2 samples × 2 treatments). Chromatic differences between treatments were evaluated using the CIELab color difference (ΔE*), corresponding to the Euclidean distance between two points (r and s) in the three-dimensional CIELab space as follows: ${∆E}^{*}={\left[{\left({∆L}^{*}\right)}^{2}+{\left({∆a}^{*}\right)}^{2}+{\left({∆b}^{*}\right)}^{2}\right]}^{0.5}$, as previously described by Pérez-Magariño et al. [20]. In addition, the L*, a*, b* values were transformed into linear red, green, blue (RGB) values. For this conversion, we used the ColorDesigner online tool [21], which implements the standard conversion algorithms.

### 2.5. Solid Phase Microextraction Gas Chromatography–Mass Spectrometry Analysis

Cheeses were grated finely with a hand-held commercial grater. A cheese sample of 2.107 ± 0.002 g was deposited into a glass vial sealed with a MilliporeSigma Supelco Mininert valve, which allowed the introduction of solid phase microextraction (SPME) needle without piercing any septa. Cheese samples were equilibrated in the sample vials for 30 min at 45 °C prior to SPME needle exposure. All extractions were carried out with a DVB/CAR/PDMS Stableflex SPME (Supelco/Sigma-Aldrich, Bellafonte, PA, USA) needle fiber, 50 µm film thickness.

An Agilent 7890 Gas Chromatograph and Agilent 5975C Mass Spectrometer (Agilent Technologies, Santa Clara, CA, USA) was used following the Faulkner et al. [14] methodology. SPME was used to sample the headspace of the cheese vial using the following procedure: Prior to the day’s analysis, the SPME needle was conditioned by exposing the needle with the inlet at 270 °C and the oven at 325 °C for 60 min. The needle was exposed to the headspace of the cheese vial for 20 min at 45 °C immediately before desorption in the gas chromatography–mass spectrometry (GC-MS) inlet. A SPME needle blank was analyzed followed by two sample runs. Another blank was then run followed again by two sample runs, and this sequence concluded with one last blank analysis. Each cheese type was analyzed four times, and the control cheese was analyzed three times under the same GC-MS parameters.

For the chromatography, a Phenomenex ZB-SemiVolatiles capillary column (30 m length × 0.25 mm ID, 0.25 µm film thickness) was used (Phenomenex, Torrance, CA, USA). The flow rate through the column was a constant 1.0 mL/min of ultrapure He carrier gas, and the column temperature was programmed as follows: (1) 40 °C for 5 min; (2) increased to 100 °C at 10 °C/min; (3). increased to 325 °C at 20 °C/min; (4) hold 10 min; total run time = 38.25 min. The mass spectromemer (MS) transfer line was kept at a temperature of 250 °C and the ion source temperature was 230 °C. The mass spectrometer scanned a *m*/*z* range used was 29–350 with a threshold of 150 counts and a scan speed of 3.125 scans per second. The source temperature was 230 °C, the quadrupole temperature was 150 °C, and the ionization energy was 70.3 eV. A standard of n-alkanes (C7-C33, Restek #31080) was analyzed by this method to construct the Kovats Retention Index (RI). An SPME alkane standard was analyzed each day and RI values for each day’s analysis was based on the alkane standard for that day. Using the above GC-MS method parameters, the C8-C25 alkanes were identified and used to create the retention indices, the retention times for the C8-C25 alkanes were approximately 4.5 to 26.5 min. Final tentatively annotated molecular features were only included if their calculated (experimental) RIs were between the C8 and C25 RI values. To perform Level 1 confirmations (see “VOC Analysis” section below), the SPME fiber was exposed to an appropriate dilution of the chemical standard in hexane in the same fashion as for the cheese samples. Spectral and RI agreement was evaluated in MS-DIAL software (v 4.9.221218), Agilent Masshunter (Qualitative Analysis v B.08.00, Quantitative Analysis v.09.00) and AMDIS (v 2.73)/NIST MS Search (v 2.4) program with visual inspection. Chemical standards for Level 1 confirmations were obtained from AA Blocks (San Diego, CA, USA).

Data files were converted to .abf files for MS DIAL (v 4.9.221218) deconvolution and spectral database matching using the Reifycs Analysis Base File Converter. The NIST 2023 EI spectral library (NIST23) was converted to an .msp library for use with MS-DIAL using the Lib2NIST program for library entries of 50,000 compounds at a time and then compiled into a single .msp (.txt) file using a large text file editor (VIM Vi Improved, v 9.0). The MS DIAL parameters for identification and alignment were the default parameters except as follows: Minimum Peak Height = 1000, Sigma Window Value = 1.0, EI Spectra Cut Off = 10, RI Identification, RI Tolerance = 20, *m*/*z* tolerance = 0.5, EI Similarity Cut Off = 70%, Identification Score Cut Off = 70%, Use Retention Information for Scoring = Yes, Use Retention Information for Filtering = Yes, RI Tolerance (alignment) = 20, EI Similarity Tolerance (alignment) = 70%, Gap Filling By Compulsion = No, Peak Count Filter = 0%. For the purposes of statistical data analysis, peak heights were normalized by the Total Ion Chromatogram (TIC) signal.

### 2.6. Sensory Evaluation

Sensory evaluation procedure was performed in accordance with the Committee for the Protection of Human Subjects that serves as the Institutional Review Board (IRB) for California State University, Fresno. Untrained panelists who were familiar with cheese consumption were selected. For screening purposes, the panelists filled out a survey where they indicated how often they consumed cheese. Participants who selected consumption of one or more times per week were included. A total of 39 panelists participated in the sensory evaluations, ranging from 21 to 50 years (mean age: 26.6). The panel consisted of 53% male, 46% female, and 1% identifying as other. The sensory test was conducted after 65 days of ripening. Each cheese was cut into triangles and weighed approximately 28 g. The panelist received four samples (two control and two wine-soaked cheeses) on a disposable white paper plate with a 3-digit randomized code adjacent to the cheese triangle, along with a water cup and unsalted cracker to clean the palate.

Panelists were required to evaluate five quality attributes: color of the rind (appearance), smell (aroma), texture, flavor, and overall liking on a 9-point line scale of rating where 1 was the lowest rating, and 9 was the highest. Additionally, the salt level was assessed using the just-about-right (JAR) scale from 1 (very little salt) to 5 (too much salt), presented as a continuous scale line, following Osunbade et al. [22] procedure. Data collection and sample presentation randomization was managed with Compusense software (Version 25.0.33398, Compusense Inc., Guelph, ON, Canada).

### 2.7. Statistical Analysis

The composition and sensory analysis of the cheese samples were conducted using Two-Way Variance Analysis (ANOVA) and Tukey’s Test [23], with a significance level set at *p* < 0.05. This analysis compared the means of four cheese samples, consisting of two wine-soaked samples and two control samples. Statistical analyses were performed using SPSS (version 29.0.1.0, IBM Corp, Santa Clara, CA, USA).

Statistical analyses for the volatiles measured by SPME-GC-MS were performed in MS Excel and MetaboAnalyst 6.0. Features were normalized by the Total Ion Chromatogram (TIC) in MS DIAL prior to analysis. Fold change analyses at a significant level of *p* < −0.01 was also performed on the TIC-normalized abundance data. The correlation heatmap was generated in MetaboAnalyst 6.0 sftware using Pearson’s R and TIC-normalized abundance data. The 85 non-contaminant alignment features were analyzed by Principal Components Analysis (PCA) after mean-centering and dividing by the standard deviation of the TIC-normalized abundance for each feature. Prior to single-factor Analysis of Variance (ANOVA), TIC-normalized abundances were log10 transformed.

## 3. Results and Discussion

**Figure 1:**
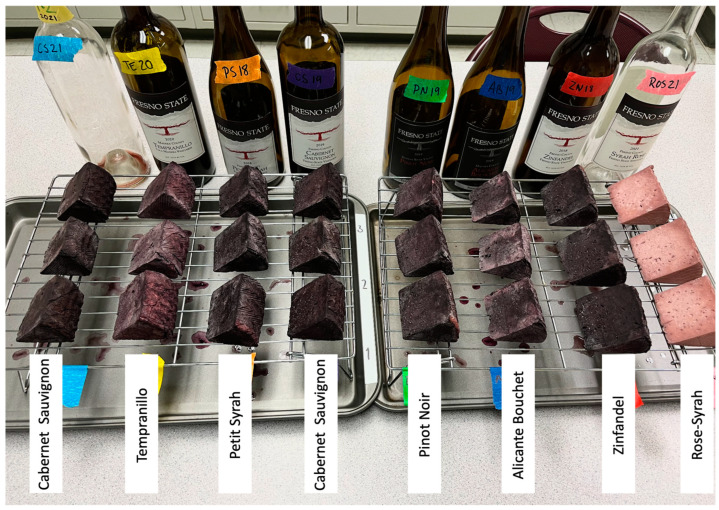
Preliminary trial to identify which selections of wine were more effective in transferring color to the cheese.

A preliminary trial was conducted to evaluate various wines, aiming to identify which selections were more effective in transferring color to the cheese. The trial used seven different red varietal wines (two Cabernet Sauvignon, Tempranillo, Petit Syrah, Pinot Noir, Alicante Bouchet, and Zinfandel) and a rosé wine (from Syrah). The results showed that not all wines were able to imprint a uniform and vividly colored rind (Figure 1). Based on rind color homogeneity, a Cabernet Sauvignon and an Alicante Bouchet wine were selected for the full chemical and microbiological characterization of the cheese. The preliminary trial also led to adjustments in the soaking process. Although an initial four-day soak was planned, uneven rind coloration was observed after two days of drying. Therefore, cheeses were re-soaked in the same wine for an additional four days, resulting in a more uniform and colored rind.

In comparison with other studies, only one research on semi-hard cheese was found that used 7 days of soaking in wine [15] but the cheese was aged first, and the homogeneity of rind color was not reported. The sequence of two soaking stages therefore was adopted as a standard method for the full characterization of the different treatments. Another study on Turkey Dil cheese (mozzarella-style) soaked the cheese in red and white wine for 28 days at 4 °C [24]. However, the significant differences in the cheesemaking process of this cheese, particularly its higher moisture and lower fat content, prevent a fair comparison with the cheese from this study. Therefore, this highlights a lack of scientific research on several key factors, such as the timing of soaking (after pressing, after initial aging, after long aging), soaking temperature, moisture content, cheesemaking process, etc., to better understand the transfer of pigments and other flavor compounds from the wine to the cheese. Using wine by-products to soak the cheese is also being studied. For example, Innocente et al. [9] aged a semi-hard Italian cheese for 8 months and then soaked it in a Merlot fermented must for 7 days. Di Cagno et al. [7] studied the Ubriaco cheese, which is a semi-hard cheese aged for 3 months in grape marc. Gaglio et al. [25] added grape pomace powder during the curd extraction, with the ripening process lasting one month.

### 3.1. Physicochemical Characteristics

Protein, fat, salt and moisture were measured as basic composition parameters. As expected, the protein and fat contents increased, while the moisture content decreased during cheese ripening at 30 and 60 days (Table 1). This occurred due to moisture loss, the dry matter increases, resulting in a higher concentration of total solids [26]. Within each ripening time, the wine-soaked cheeses showed significantly higher protein and fat percentages than their time-matched controls cheese samples (*p* < 0.05), which means more moisture content in wine-soaked cheeses than in no-soaked cheeses (Table 1). However, lower moisture was expected in wine-soaked cheeses, as the ethanol content and low pH of wine can create an osmotic gradient that promotes dehydration. A significant decrease of 3.3% in salt content (*p* = 0.04) was observed in both of the wine-soaked treatments at both 30 and 60 days (Table 1). A potential reason for the decrease in salt content is the fact that the wine used for soaking did not contain any salt and generated a concentration gradient that encouraged the migration of salt from the cheese to the wine to restore osmotic pressure equilibrium [27]. This drop in salt percentage in the wine-soaked samples provided a way to equilibrate the salt content to values that are aligned with similar semi-hard cheeses that range from 1.5 to 2.5% [28,29]. Guler et al. [24] found a similar decrease in salt content for their red and white wine soaked Italian Dil cheese.

**Figure 2:**
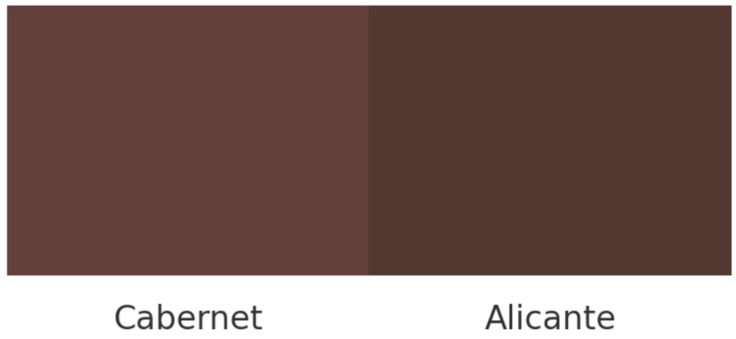
Comparation of colors between the Cabernet Sauvignon and the Alicante Bouchet treatment.

The color coordinate values L*, a*, b* shown in Table 2 for the wine-soaked treatments clearly demonstrate differences among the treatments, particularly in the higher luminosity (L*) and lower b* coordinate (blue to yellow) values in the Cabernet Sauvignon treatment. In fact, the total difference from the combined coordinates, evaluated through Delta E (ΔE*)*,* is 5.45, which clearly suggests that a large color difference exists between the samples. A conversion into Red, Green, Blue (RGB) values is indicative of the degree of difference, where the Cabernet treatments had a lighter red-brown-mauve color compared to the Alicante Bouchet treatment with a darker purple tone (Figure 2). Cabernet Sauvignon and Alicante Bouschet wines are both known for having a naturally high concentration of pigments (anthocyanins) in comparison to the other varietal wines used in the preliminary test. In fact, Alicante Bouschet wine is known as one of the most highly pigmented varietal wines [30]. The higher b* value in Alicante Bouschet cheese reflects a more pronounced reddish-yellow tone, consistent with the higher anthocyanin and phenolic content typically reported for this varietal [31]. The a* values did not differ significantly, suggesting similar red intensity across treatments. Overall, the darker and more saturated color in Alicante Bouschet cheeses supports the greater pigment diffusion from this wine and may also indicate higher phenolic transfer, which could contribute to mild antimicrobial effects at the rind surface and influence mold development and sensory perception [32].

### 3.2. Microbiology

The TPC, total coliforms, yeasts and molds, and *Listeria* numbers of cheese samples are shown in Table 3, the two control samples were combined since no statistical differences were found. In general, the TPC decreased during the time on all cheese samples, reflecting a typical and desirable trend after the ripening period. Similar observations have been reported by Licón et al. [19], who documented reductions in all microbiological groups studied during cheese aging. The lowest TPC was 2.0 × 10^4^ cfu g^−1^ in the Cabernet-soaked cheese. Previously, Cabezas et al. [33] reported about 2.0 × 10^8^ cfu g^−1^ on the TPC after 60 days of ripening in artisanal Manchego cheeses manufactured from ovine raw milk. Poveda et al. [34] reported that the TPC count in Manchego cheese produced with pasteurized milk was approximately 3.4 × 10^8^ cfu g^−1^. There was a decline in coliform counts during ripening. It has been studied that soaking cheese in wine may reduce harmful bacteria, enhancing microbiological safety [8]. Upon reaching the 60 days, the cheeses reached the minimum microbial limit authorized (≤100 cfu g^−1^) [35]. The reduction in microorganism concentrations observed in wine-soaked cheeses can be attributed to the antimicrobial properties of wine constituents such as ethanol, organic acids and phenolic compounds, including tannins and anthocyanins [8,32,36]. Phenolic compounds can inhibit microorganisms by binding to their cell membranes, interfering with their enzymes, and depriving them of essential nutrients and metal ions [32]. Additionally, ethanol and organic acids decrease the surface pH and water activity, making it harder for microbes to grow [37]. The yeast and molds count of both wine-soaked cheeses after the 60 days of ripening time was around 5.0 × 10^3^ cfu g^−1^. Although, no visible fungal growth was detected throughout the aging process. Mold development on the rind of Manchego cheese is a common sensory defect that may lead to economic losses [38]. However, sometimes mold is allowed to grow during the first weeks of ripening to improve the flavor and the consolidation of the rind. Subsequently, an antifungal coating is applied to prevent further fungal growth throughout the remaining aging period [39]. This step could be easily implemented in a commercial operation. The *Listeria* analysis gave no colony forming counts across all days as expected.

### 3.3. Volatile Compounds

The MS-DIAL analysis performed on the SPME-GC-MS data produced 381 molecular features (alignment spots). MS-DIAL classified 153 of these spots as Reference Matched based on the criteria described in the SPME–GC–MS analysis section above.

The alignment spots in MS-DIAL were manually inspected to combine redundant or split features where necessary. This process generated a total of 377 features where 104 were discarded due to poor peak shape or low signal to noise throughout visual inspection. Of the remaining 273 valid features, 123 were initially in the reference matched group, and of those 123, 11 were reclassified as unknown since they were outside the alkane RI window (C8, 4.527 min–C23, 25.684 min), 8 were reclassified as unknown because they were below the annotation threshold of more than one replicate within a class (Alicante/Cabernet/control) or 80% overall, and 39 were reclassified as unknown based on manual inspection (unlikely to be present in cheese headspace). This left a final data set of 273 features, 65 tentative identifications, and 208 unknowns. Each alignment spot was manually inspected and integrated if necessary in MS-DIAL.

These 273 features were then tested against the 1-sided 99% confidence interval of all the SPME blanks analyzed (*n* = 9). Features in which the mean TIC-corrected abundance was less than this confidence interval threshold were classified as contaminants and eliminated from further analysis. This analysis eliminated 188 features and left 85 features (42 tentative identifications, 43 unknowns). A subset of these annotations was confirmed by analytical standards, e.g., Level 1 confidence (indicated in Table 4 by the asterisk superscript) while the remainder are tentative annotations corresponding to Level 2 confidence on the scale proposed by Schymanski, et al. (2014) [40]. A total of 16 analytical standards were analyzed, and the initial Level 2 identifications (e.g., moved to Level 1) were confirmed in 14 of the 16. The two that were not confirmed, with Level 2 identifications of dodecane and benzyl alcohol, were reclassified as “Unknowns” and added to Table 5 as Unknown 2001 and 2002, respectively. As such, the final tabulation of unknowns, Level 2, and Level 1 identification was: 85 total features, 14 Level 1 identifications, 26 Level 2 identifications, and 45 unknowns. Table 4 provides a summary of the tentative identifications categorized by nominal chemical class. This table provides the measured alkane-based RI value that could be compared with the expected RI value for that chemical based on the NIST 2023 database. In addition, it presents the *m*/*z* value used for abundance determination, (specifically, the ‘quant mass’ from MS-DIAL) and the mean TIC-corrected abundances for each cheese type. Furthermore, the *p*-value and Tukey’s Honest Significant Difference (HSD) post hoc test result from an Analysis of Variance (ANOVA) test are provided.

Table 4 shows many commonly reported volatiles associated with the headspace analysis of cheese were present. Ethyl esters of free fatty acids ranging from C8 to C12 and then C16-C18 were present, with six of them (acetic acid 2 phenyl ester, octanoic acid ethyl ester, tetradecanoic acid ethyl ester, decanoic acid ethyl ester, nonanoic acid ethyl ester, and ethyl 9-decenoate) being enriched in the Cabernet-soaked cheese in comparison to the Alicante-soaked cheese. A comparison of the compositional analysis (Table 1) with the VOC data indicates that the higher moisture and lower fat content Cabernet Sauvignon may be related to the higher phenylethylalcohol, 2-phenylethyl acetic acid, and benzeneacetaldehyde VOC content in the Cabernet-soaked cheese.

Many of the volatile compounds identified in the headspace analysis were also measured in HS-SPME-GC-MS analyses of wines. Gürbüz et al. [41] used HS-SPME-GC-MS and GC-Olfactometry analysis to investigate Cabernet Sauvignon and Merlot wines from USA and Australia. Several volatiles found in Cabernet Sauvignon were also identified in our study, including phenylethyl alcohol, octanoic acid ethyl ester, decanoic acid ethyl ester, and 2-phenylethyl ester. In a more recent study, de Macêdo Morais et al. [42] identified phenylethyl alcohol, many esters, such us octanoic acid ethyl ester, nonanoic acid ethyl ester acetic acid, 2-phenylethyl ester, and 1-butanol 3 methyl acetate. In the same research, phenylethyl alcohol and acetic acid 2-phenylethyl ester were either more prevalent in Alicante wine compared to Cabernet Sauvignon (for phenylethyl alcohol) or present in similar amounts (for 2-phenylethyl ester). However, in our study, these VOCs were found to be significantly enriched in the Cabernet-soaked cheese, although the fold changes for both were less than 3. Additionally, 1-butanol 3-methyl acetate was slightly enriched in our Alicante-soaked cheese, while nominally equivalent amounts of this VOC were reported in both wines [42]. Moreover, de Macêdo Morais et al. [42] found that in phenylethyl alcohol and 2-phenylethyl ester were correlated with each other.

Data on the 45 features classified as “Unknown” are presented in Table 5. Although these compounds have not yet been fully identified, their retention indices (RI) and *m*/*z* values suggest that many may belong to the ester, ketone, or aldehyde families, which are known contributors to cheese aroma and ripening dynamics. Of these 45 Unknowns, eight were found to be significantly enriched in the Cabernet-soaked cheese in comparison with the Alicante-soaked cheese. The quantifier *m*/*z* values for these eight compounds did not show any apparent pattern and were not constrained to one value (meaning, the mass spectra of these Unknowns did not have a single prevailing dominant ion). Unknown 185 was also enriched in the Cabernet-soaked cheese, with the quantifier ion of *m*/*z* 88 indicates a likely ethyl ester functionality; however, Unknown 171 also has a quantifier ion of *m*/*z* 88 although it is not enriched in any of the cheese types.

**Figure 3:**
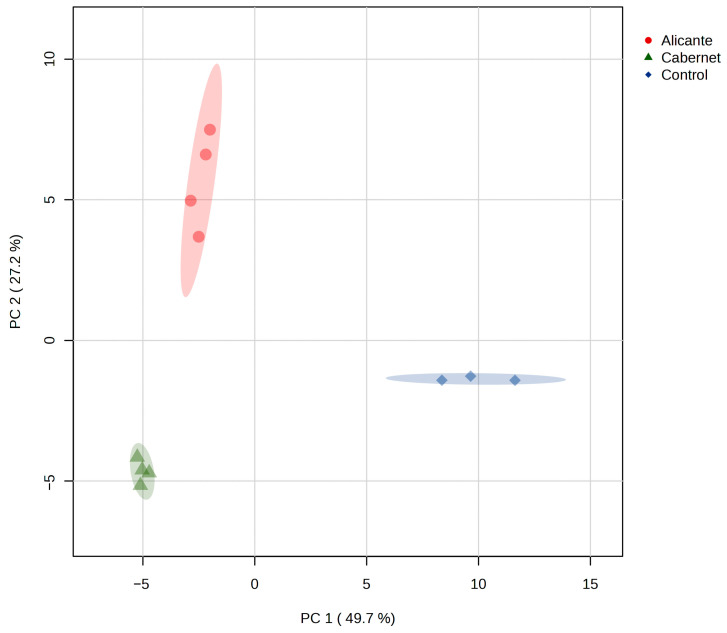
Principal Components Analysis Scores plot showing grouping of three cheese categories on Principal Component 1 (PC1) and Principal Component 2 (PC2).

A Principal Components Analysis (PCA) was performed to assess the overall grouping of the volatile compounds, including both identified and unidentified compounds (Figure 3). The analysis revealed a notable tight clustering among the different cheese types, with principal components 1 and 2 accounting for over 76% of the total variance. When the third dimension of the PCA was added, an additional 13.4% variance was explained, increasing the total variance explained to 90%. As shown in the scores plot, the Cabernet and Alicante-soaked cheese are primarily similar along PC1, and both differ from the control cheese along this axis. Moreover, the Cabernet and Alicante cheeses differ from each other primarily along PC2.

**Figure 4:**
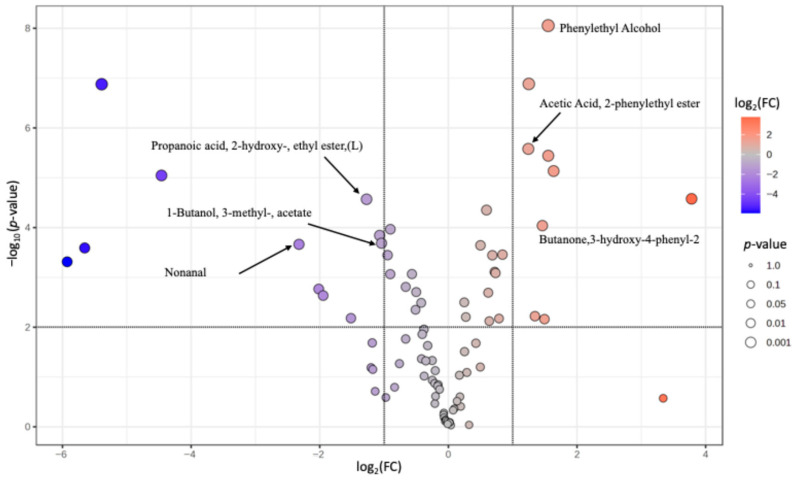
Volcano plot representing log2 of fold change (FC) of TIC-corrected mean abundance in Cabernet-soaked cheese compared to Alicante-soaked cheese on *x*-axis versus log10 of *p*-value from an unpaired *t*-test for means assuming equal variance. The horizontal lines represent an FC threshold of 2 and the vertical lines represent a raw *p*-value threshold of 0.01. Only tentatively identified features are noted.

A volcano plot for the ratio of the Cabernet cheese TIC corrected mean with Alicante cheese TIC corrected mean is shown in Figure 4. Phenylethyl alcohol, acetic acid 2 phenylethyl ester, and 3-hydroxy-4-phenyl-2-butanone were all significantly enriched in the Cabernet cheese as compared with the Alicante cheese, while nonanal, 3-methyl-1 butanol acetate and 2 hydroxy propanoic acid, ethyl ester were significantly enriched in the Alicante cheese relative to the Cabernet cheese.

The volatile component phenylethyl alcohol was enriched in both the Cabernet-soaked (Cabernet: control Fold Change (FC) = 15.8) and Alicante-soaked cheese in comparison to the control (Alicante: control FC = 5.4), but this enrichment was far greater in the Cabernet-soaked cheese (Cabernet: Alicante FC = 2.9). All three of these enrichments were significant (based on an unpaired *t*-test at *p* < 0.01). In general, there are predominant number of esters compounds, which are associated with pleasant fruity and floral aromas, such as acetic acid, 2-phenylethyl ester, butanedioic acid, diethyl ester and propanoic acid, 2-hydroxy-, ethyl ester [43,44], that contribute to desirable flavor notes in ripened cheeses. Their higher concentrations in the wine-soaked samples, particularly in the Cabernet treatment, suggest that wine components enhanced ester formation or diffusion, favoring positive aromatic attributes. In addition, the ester 2-phenylethyl acetate (acetic acid 2-phenyl ethyl ester) was also enriched in the Cabernet-soaked cheese in comparison to both the control (Cabernet:control FC = 7.7) and Alicante-soaked cheeses (Cabernet: Alicante FC = 2.4), both also *p* < 0.01. Carunchia et al. [45] describe the generation of phenylethyl alcohol, and phenylethyl acetate, phenyl acetic acid, and benzeneacetaldehyde as being the result of the Strecker degradation of aromatic amino acids (primarily phenylalanine but also tryptophan and tyrosine). In wine, these compounds are primarily produced by yeast amino acid metabolism during alcoholic fermentation, and many of them are present at suprathreshold concentrations that contribute to the general fermentation aroma present in all wines [46]. Phenylethyl alcohol and 2-phenyl ethyl acetate are described as being responsible for a floral aroma, and as potentially contributing to the final flavor of raw milk camembert [47]. These two volatile compounds are associated with a rosy, floral aroma in cheeses [45,47] or wines [48]. Phenylethyl alcohol was found in cheeses at the end of the ripening process of cheese produced by lactic acid bacteria by Li et al. [49]. The ketone 3-hydroxy-4-phenyl-2-butanone has been identified in wine and apple cider vinegar and described to have a floral and clove-like odor [50].

Of the compounds found to be enriched in Alicante as compared with Cabernet, the aliphatic aldehyde nonanal is commonly found in mozzarella cheeses and is classified as having a grassy odor [51], whereas in wine is described as citrus [52]. In wine, aldehydes such as this are typically formed by oxidation of higher alcohols [46], and it is likely that this and other aldehydes from higher alcohols increase during cheese maturation due to the oxidative conditions inherent to the process. The ethyl ester of 2-hydroxy propanoic acid (ethyl lactate) is generally present in wines that undergo malolactic fermentation by lactic acid bacteria; while in wine this volatile compound tends to be of low sensory relevance due to its low volatility in the presence of alcohol [46,53], although in Baijiu, a Chinese spirit, it has been shown to contribute to fruity aromas [54]. The ester 3-methyl-1-butanol acetate (isoamyl acetate) is usually found in wines at suprathreshold concentrations and contribute to the general fruitiness and banana aroma that is ubiquitous in young wines [55].

**Figure 5:**
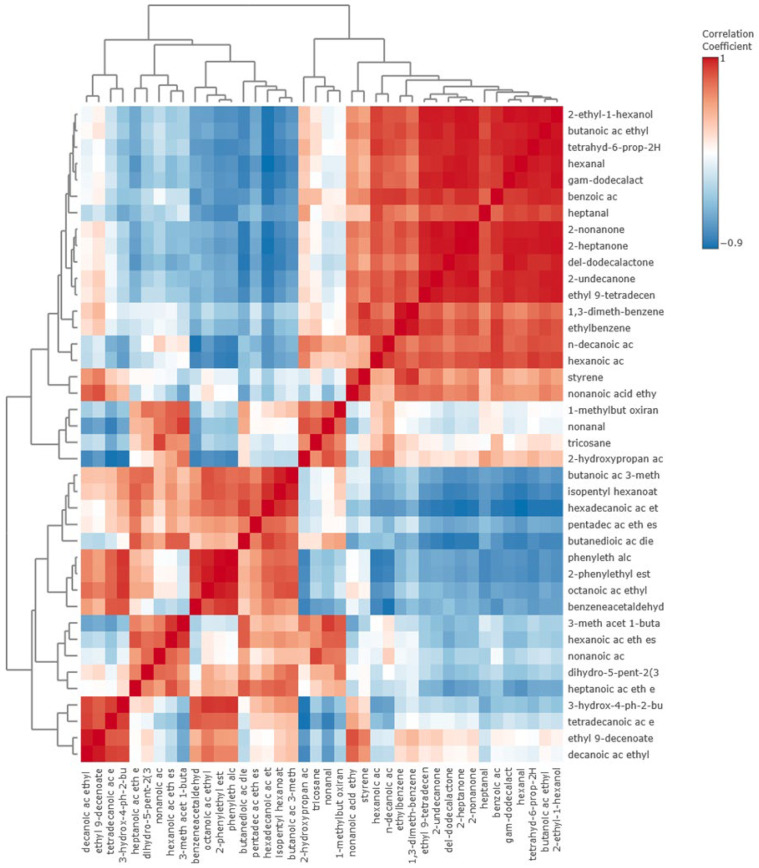
Correlation heatmap, with clustering to group similarly varying features, with abbreviated VOC names. Correlation coefficient was calculated as Pearson’s R.

Figure 5 presents a correlation heatmap of the tentatively identified and confirmed VOCs to help visualize the relationship between VOCs (Pearson’s R). Many of the ethyl esters are well-correlated, as well as the phenylethylalcohol, 2-phenylethyl acetic acid, and benzeneacetaldehyde VOCs discussed above as likely being generated by the Strecker degradation of aromatic amino acids. Ketone and aldehyde groups were not observed to vary in concert with the ethyl esters. In addition, esters such as ethyl butyrate, ethyl hexanoate, ethyl octanoate, ethyl decanoate, and 2-phenylethyl acetate showed strong positive correlations (r > 0.8) with one another. These esters are responsible for fruity and floral aromas, consistent with the sensory preference observed for this treatment. Similarly, aromatic alcohols (such as 2-phenylethanol) were positively correlated with these esters, suggesting that wine-derived precursors and fermentation processes enhanced the production of pleasant, sweet notes.

These differences clearly show that each wine was able to provide a distinctive volatile profile to the cheese. In general, the main difference between Cabernet Sauvignon and Alicante Bouschet wine varieties is the concentration and composition of volatile compounds and polyphenols including anthocyanins and tannin. Cabernet Sauvignon is known for having a high concentration of tannins (1 to 2.3 g/L [56]), which are largely responsible for astringency in red wine. In addition, Cabernet Sauvignon is known for its high concentration of pyrazines (33 to 36 ng/L, threshold 0.3 to 2 ng/L [46]), such as isobutyl methoxypyrazine and isopropyl methoxypyrazine, which are responsible for green pepper or vegetal aromas. Cabernet Sauvignon is known as an international varietal due to its widespread adoption throughout the main winemaking regions of the world, covering 341,000 ha or 4% of the world’s vineyards [57]. On the other hand, Alicante Bouschet is frequently used as a source of red color for wine blends due to its high concentration of anthocyanins (800 to 1600 mg/L [30]) which often represents three times the typical concentration of other red wine varietals.

### 3.4. Sensory Evaluation Results

**Figure 6:**
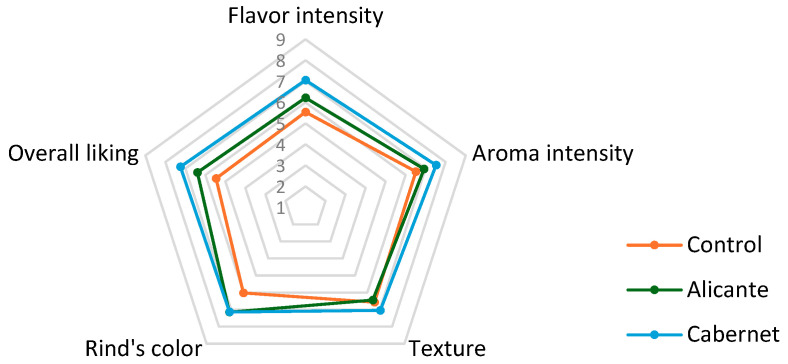
This is a figure. Sensory profile of cheeses soaked in two different red wines using 9-point hedonic scales. Mean scores from *n* = 39 panelists.

The sensory panel (*n* = 39, female 45%, mean age = 26.6 years old) evaluated five quality attributes: color of the rind (appearance), smell (aroma), texture, flavor, and overall liking one sample at time. The sensory profile for the cheese treatments is shown as a radial diagram in Figure 6. Significant differences were found (*p* < 0.01) between cheeses soaked in wine and control cheeses for flavor, overall liking, and color of rind attributes (Table 6). These results clearly show that the choice of soaking wine can have an impact on sensory perception, particularly in acceptance of the product. As expected, there were no significant differences in texture between treatments. The saltiness was also evaluated, which was measured using a just-about-right scale. Control cheeses received an average rating of 4.2 corresponding to the “extremely salty” side of the scale (ranging from 1 to 5 where 3 indicates Just-about-right), and therefore a product out of balance. In contrast, both wine-soaked cheeses obtained a score of 2.9 (just-about-right), indicating an appropriate level of salt, and in agreement with typical values for semi-hard cheeses [58]. This result also evidenced that the soaking process was able to remove excess salt by creating a gradient of concentration where salt migrated from the cheese to the wine, allowing to balance the sensory profile and likely the acceptability of the final product. This result also brings to attention how the brining process would need to be optimized so that after wine soaking, the salt level is well liked by consumers. It is possible that adding salt to the wines before soaking could be used as a strategy to minimize the desalination of cheeses and, therefore, limit drastic changes in the flavor balance.

## 4. Conclusions

A two-stage soaking method (4 days soak, 2 days dry, 4 days re-soak) produced a uniform rind coloration, with Cabernet Sauvignon wine imparting lighter red-brown tones and Alicante Bouschet wine creating darker purple hues. While fat, protein, and moisture contents were unaffected, a significant reduction in salt (≈3.3%) occurred in wine-soaked cheeses, resulting in levels consistent with typical semi-hard cheeses and enhancing sensory balance. Microbiological analyses demonstrated that the soaking process neither compromised the microbiological stability nor induced atypical aging trajectory of the cheese. Volatile profiling revealed a clear differentiation between treatments, with Cabernet-soaked cheeses displaying floral volatiles (e.g., phenylethyl alcohol, 2-phenylethyl acetate) and Alicante-soaked cheeses showing more fruity volatiles (e.g., nonanal, isoamyl acetate). Sensory evaluation confirmed greater consumer acceptance of wine-soaked cheeses over controls, highlighting wine soaking as a viable method for producing distinctive, well-liked artisanal cheeses. Exploring different wine varieties, soaking durations, and cheese types can significantly enhance the wine-soaking process and inspire innovative artisanal products. Additionally, studying the microbial dynamics during soaking and ripening will reveal how wine components influence flavor and texture, paving the way for exciting advancements in the industry.

## Figures and Tables

**Table 1:** Composition analysis of the control (C), Cabernet Sauvignon (CS), and Alicante Bouchet (AB)-soaked cheeses aged for 30 and 60 days.

Day	Protein (%)	Fat (%)	Salt (%)	Moisture (%)
Start				
C_CS_	15.2 ± 0.8 ^a^	20.0 ± 0.5 ^a^	4.4 ± 0.1 ^a^	51.8 ± 0.2 ^a^
C_AB_	17.2 ± 0.8 ^b^	22.5 ± 0.0 ^b^	2.9 ± 0.2 ^b^	50.5 ± 0.4 ^a^
Day 30				
C_CS_	17.8 ± 0.0 ^a^	23.2 ± 0.2 ^a^	5.7 ± 0.0 ^b^	44.3 ± 0.4 ^b^
C_AB_	19.4 ± 0.1 ^b^	25.4 ± 0.1 ^b^	5.8 ± 0.0 ^b^	40.7 ± 0.0 ^a^
CS	20.4 ± 0.1 ^c^	23.1 ± 0.2 ^a^	2.3 ± 0.0 ^a^	46.2 ± 0.0 ^c^
AB	22.1 ± 0.2 ^d^	25.4 ± 0.9 ^b^	2.2 ± 0.0 ^a^	43.1 ± 0.8 ^b^
Day 60				
C_CS_	20.6 ± 0.0 ^b^	27.0 ± 0.1 ^c^	5.5 ± 0.1 ^b^	38.7 ± 0.1 ^a^
C_AB_	18.1 ± 0.2 ^a^	22.9 ± 0.2 ^a^	5.4 ± 0.2 ^b^	45.1 ± 0.5 ^c^
CS	21.3 ± 0.1 ^c^	24.8 ± 0.1 ^b^	2.2 ± 0.0 ^a^	44.4 ± 0.2 ^b,c^
AB	21.3 ± 0.1 ^c^	25.0 ± 0.1 ^b^	2.2 ± 0.0 ^a^	43.8 ± 0.2 ^b^

Mean values ± standard deviation; ^a–c^: values with different superscript per column and day were significantly different (*p* < 0.05).

**Table 2:** Color coordinates from cheeses soaked in wine according to the Commission Internationale d’Eclairage (CIE) L*, a* and b* after 60 days of ripening. A conversion to RGB representation is given for reference.

	L*	a*	b*	Red	Green	Blue
Cabernet Sauvignon	36.13 ± 3.13 ^a^	12.95 ± 1.06 ^a^	1.95 ± 1.04 ^a^	106 ± 10 ^a^	77 ± 6.5 ^a^	82 ± 5.5 ^a^
Alicante Bouchet	30.91 ± 7.88 ^a^	12.87 ± 3.50 ^a^	3.53 ± 1.44 ^b^	95 ± 25 ^a^	65 ± 15.5 ^a^	66 ± 16 ^a^

Mean values ± standard deviation; ^a,b^: values with different superscript per column and day were significantly different (*p* < 0.05).

**Table 3:** Microbial counts (cfu/g) from cheese samples.

Day	TPC	Coliforms	Yeast and Mold	Listeria
Start				
C	5.2 × 10^8^	6.5 × 10^3^	1.9 × 10^4^	(-)
Day 30				
C	9.3 × 10^6^	5.0 × 10^2^	1.4 × 10^4^	(-)
CS	2.6 × 10^4^	3.2 × 10^1^	7.6 × 10^4^	(-)
AB	5.3 × 10^6^	1.3 × 10^2^	7.6 × 10^3^	(-)
Day 60				
C	3.3 × 10^4^	<	2.8 × 10^4^	(-)
CS	2.0 × 10^4^	<	5.0 × 10^3^	(-)
AB	2.0 × 10^6^	<	4.8 × 10^3^	(-)

Mean values of two repetitions, except for control samples, which are based on four replicates: two from the control cheese of Cabernet-soaked cheese, and two from the control cheese of Alicante-soaked cheese; TPC: total plate count, C: control, CS: Cabernet Sauvignon-soaked cheese, AB: Alicante Bouchet-soaked cheese; <: too few to count.

**Table 4:** Volatile compounds identified by headspace solid-phase micro extraction GC-MS analysis of features in Cabernet Sauvignon-soaked, Alicante Bouchet-soaked, and control cheese headspace.

Compound	CAS	*m*/*z*	Experimental Mean RI	NIST Median RI	Control Mean	Alicante Mean	Cabernet Mean	*p*-Value
			Mean SD = 0.46	Mean SD = 3.79	95% CI of %RSD: 17 ± 5	95% CI of %RSD: 14 ± 5	95% CI of %RSD: 16 ± 4	
Esters (*n* = 16)								
**Acetic acid, 2-phenylethyl ester^@^**	**103-45-7**	**104**	**1262**	**1258**	**1.75 × 10^−4^** ^a^	**5.69 × 10^−4^** ^b^	**1.35 × 10^−3^** ^c^	**1.90 × 10^−8^**
Butanedioic acid, diethyl ester^@^	123-25-1	101	1188	1181	4.43 × 10^−4 **a**^	3.29 × 10^−3 b^	2.31 × 10^−3 c^	9.23 × 10^−8^
Hexadecanoic acid, ethyl ester^@^	628-97-7	88	1994	1993	5.85 × 10^−4 a^	1.33 × 10^−3 a,b^	1.40 × 10^−3 b^	2.15 × 10^−6^
Propanoic acid, 2-hydroxy-,ethyl ester, (L)^@^	687-47-8	45	813	848 *	5.26 × 10^−3 a^	6.31 × 10^−3 a^	2.61 × 10^−3 b^	3.56 × 10^−6^
**Octanoic acid, ethyl ester^@^**	**106-32-1**	**88**	**1198**	**1196**	**7.91 × 10^−3^** ^a^	**1.16 × 10^−2^** ^b^	**2.08 × 10^−2^** ^c^	**4.80 × 10^−5^**
Butanoic acid, ethyl ester^@^	105-54-4	71	803	802	3.90 × 10^−3 a^	1.65 × 10^−3 a,b^	1.44 × 10^−3 b^	6.16 × 10^−5^
**Tetradecanoic acid, ethyl ester**	**124-06-1**	**88**	**1794**	**1793**	**2.96 × 10^−3^** ^a^	**2.57 × 10^−3^** ^b^	**3.64 × 10^−3^** ^c^	**0.00013**
Ethyl 9-tetradecenoate	24880-50-0	55	1783	1787 *	1.59 × 10^−4 a^	1.06 × 10^−4 a,b^	1.05 × 10^−4 b^	0.00027
Isopentyl hexanoate^@^	2198-61-0	70	1255	1250	9.20 × 10^−5 a^	1.62 × 10^−4 a,b^	1.77 × 10^−4 b^	0.00053
**Decanoic acid, ethyl ester**	**110-38-3**	**88**	**1395**	**1396**	**1.63 × 10^−2^** ^a^	**1.23 × 10^−2^** ^b^	**1.98 × 10^−2^** ^a^	**0.00062**
Butanoic acid, 3-methylbutyl ester^@^	106-27-4	70	1059	1056	1.26 × 10^−4 a^	2.26 × 10^−4 a,b^	2.58 × 10^−4 b^	0.00133
**Nonanoic acid, ethyl ester**	**123-29-5**	**88**	**1296**	**1295**	**1.12 × 10^−3^** ^a^	**6.05 × 10^−4^** ^b^	**9.40 × 10^−4^** ^a^	**0.00294**
**Ethyl 9-decenoate**	**67233-91-4**	**55**	**1388**	**1388**	**3.13 × 10^−4^** ^a^	**2.34 × 10^−4^** ^b^	**3.59 × 10^−4^** ^a^	**0.00465**
Hexanoic acid, ethyl ester	123-66-0	88	1001	999	4.77 × 10^−3 a^	7.17 × 10^−3 a^	5.51 × 10^−3 a^	0.01245
Heptanoic acid, ethyl ester^@^	106-30-9	88	1101	1098	7.72 × 10^−5 a^	1.68 × 10^−4 a^	1.46 × 10^−4 a^	
Pentadecanoic acid, ethyl ester	41114-00-5	88	1894	1894	6.49 × 10^−5 a^	7.77 × 10^−5 a^	7.86 × 10^−5 a^	
Acids (*n* = 4)								
Hexanoic acid	142-62-1	60	988	990	2.65 × 10^−3 a^	1.98 × 10^−3 b^	1.34 × 10^−3 c^	0.00011
Benzoic acid	65-85-0	105	1171	1177	1.91 × 10^−3 a^	7.40 × 10^−4 a,b^	4.36 × 10^−4 b^	0.00045
n-Decanoic acid	334-48-5	60	1365	1372	2.19 × 10^−3 a^	1.67 × 10^−3 a^	1.06 × 10^−3 b^	0.00203
Nonanoic acid^@^	112-05-0	60	1273	1273	1.84 × 10^−3 a^	6.48 × 10^−3 a^	3.30 × 10^−3 a^	
Alcohols (*n* = 2)								
**Phenylethyl Alcohol^@^**	**60-12-8**	**91**	**1115**	**1116**	**1.69 × 10^−2^** ^a^	**9.10 × 10^−2^** ^b^	**2.67 × 10^−1^** ^c^	**7.67 × 10^−1^**
1-Hexanol, 2-ethyl-	104-76-7	57	1031	1030	4.37 × 10^−4 a^	1.88 × 10^−4 b^	1.50 × 10^−4 c^	1.59 × 10^−6^
Ketones (*n* = 9)								
2-Heptanone	110-43-0	43	892	891	3.45 × 10^−3 a^	1.79 × 10^−4 b^	1.35 × 10^−4 c^	6.06 × 10^−1^
2-Nonanone	821-55-6	58	1093	1092	1.32 × 10^−3 a^	2.81 × 10^−4 a,b^	3.17 × 10^−4 b^	7.24 × 10^−8^
2H-Pyran-2-one, tetrahydro-6-propyl-	698-76-0	99	1287	1288	1.64 × 10^−4 a^	8.33 × 10^−5 b^	6.21 × 10^−5 c^	1.24 × 10^−6^
.delta.-Dodecalactone	713-95-1	99	1721	1720	4.64 × 10^−4 **a**^	1.53 × 10^−4 a,b^	1.73 × 10^−4 b^	9.51 × 10^−6^
2-Undecanone	112-12-9	58	1295	1294	5.63 × 10^−4 **a**^	2.54 × 10^−4 a,b^	2.71 × 10^−4 b^	1.88 × 10^−5^
**Butanone,3-hydroxy-4-phenyl-2-**	**5355-63-5**	**91**	**1353**	**1361** **	**5.30 × 10^−5^** ^a^	**4.62 × 10^−5^** ^a^	**1.27 × 10^−4^** ^b^	**2.55 × 10^−5^**
1-Butanol, 3-methyl-, acetate^@^	123-92-2	43	880	876	5.26 × 10^−4 a^	1.23 × 10^−3 b^	5.96 × 10^−4 a^	4.41 × 10^−5^
.gamma.-Dodecalactone	2305-05-7	85	1690	1681	2.92 × 10^−4 a^	1.42 × 10^−4 a^	1.29 × 10^−4 a^	
2(3H)-Furanone, dihydro-5-pentyl-	104-61-0	85	1368	1365	2.07 × 10^−4 a^	2.45 × 10^−4 a^	2.37 × 10^−4 a^	
Aldehydes (*n* = 4)								
Hexanal	66-25-1	56	803	801	9.24 × 10^−4 a^	1.72 × 10^−4 b^	8.95 × 10^−5 c^	1.35 × 10^−8^
Nonanal *	124-19-6	57	1108	1104	7.56 × 10^−4 a^	1.78 × 10^−3 b^	3.57 × 10^−4 c^	8.62 × 10^−5^
**Benzeneacetaldehyde^@^**	**122-78-1**	**91**	**1047**	**1045**	**4.22 × 10^−4^** ^a^	**5.12 × 10^−4^** ^a^	**8.84 × 10^−4^** ^b^	**0.00209**
Heptanal	111-71-7	70	905	901	1.82 × 10^−4 a^	9.35 × 10^−5 a^	4.25 × 10^−5 a^	0.01641
Miscellaneous (*n* = 5)								
Ethylbenzene	100-41-4	91	859	855	4.87 × 10^−4 a^	3.11 × 10^−4 a,b^	2.96 × 10^−4 b^	0.01840
Benzene, 1,3-dimethyl-	108-38-3	91	868	866	4.13 × 10^−4 a^	2.92 × 10^−4 a^	2.97 × 10^−4 a^	
Oxirane, (1-methylbutyl)-	53229-39-3	56	870	743 *	1.01 × 10^−3 a^	1.25 × 10^−3 a^	9.32 × 10^−4 a^	
Styrene	100-42-5	104	890	893	9.56 × 10^−4 a^	6.61 × 10^−4 a^	8.07 × 10^−4 a^	
Tricosane	638-67-5	57	2300	2300 ***	2.79 × 10^−4 **a**^	3.71 × 10^−4 a^	1.64 × 10^−4 a^	

Mean values; ^a–c^: values with different superscript per row were significantly different (*p* < 0.05); *p*-value: significance of one-way ANOVA test; @: Identity confirmed with analytical standard (e.g., Level 1 confirmation); **Bolded** rows: features that are significantly enriched in the Cabernet-soaked cheese compared to the Alicante-soaked cheese; RI value is definitional; #NIST RI Values are Median (Median Average Deviation): values for semi-standard non-polar column entries in the 2023 NIST library; The average standard deviations for the experimental RI values for the tentative identifications = 0.46 and the median average deviations for the NIST RI Values = 3.79; *: estimated non-polar retention index (n-alkane scale) from the NIST 2023 library entry; **: average and SD from NIST Webbook normal alkane RI, non-polar column, temperature ramp; ***: normal alkane RI, non-polar column, custom temperature program.

**Table 5:** Volatile compounds unidentified by headspace solid-phase micro extraction GC-MS analysis of features in Cabernet Sauvignon-soaked, Alicante Bouchet-soaked, and control cheese headspace.

Unknown	*m*/*z*	Experimental Mean RI	Control Mean	Alicante Mean	Cabernet Mean	*p*-Value
			95% CI of %RSD: 21 ± 5	95% CI of %RSD: 17 ± 4	95% CI of %RSD: 29 ± 9	
Unknown 41	105	677	4.70 × 10^−4^	4.43 × 10^−4^	3.83 × 10^−4^	
Unknown 42	55	687	1.76 × 10^−2^	2.22 × 10^−2^	2.29 × 10^−2^	
Unknown 44	57	692	4.14 × 10^−3^	5.69 × 10^−3^	5.50 × 10^−3^	
Unknown 52	45	772	6.26 × 10^−3^	2.74 × 10^−3^	3.43 × 10^−3^	
Unknown 60	55	804	3.90 × 10^−4^	1.52 × 10^−4^	1.28 × 10^−4^	3.13 × 10^−6^
Unknown 67	60	840	1.91 × 10^−4^	1.50 × 10^−4^	1.30 × 10^−4^	0.005286
Unknown 74	91	867	3.02 × 10^−4^	2.40 × 10^−4^	2.29 × 10^−4^	
Unknown 78	87	880	6.57 × 10^−5^	1.54 × 10^−4^	7.32 × 10^−5^	1.32 × 10^−5^
Unknown 79	43	883	1.54 × 10^−4^	2.49 × 10^−4^	1.10 × 10^−4^	0.021946
Unknown 81	91	891	2.14 × 10^−4^	1.46 × 10^−4^	1.42 × 10^−4^	
Unknown 83	85	895	5.87 × 10^−5^	1.59 × 10^−5^	3.94 × 10^−6^	0.000195
Unknown 85	45	905	2.12 × 10^−4^	1.27 × 10^−4^	6.78 × 10^−5^	7.01 × 10^−7^
**Unknown 86**	**106**	**969**	**6.38 × 10^−4^**	**3.87 × 10^−5^**	**1.09 × 10^−4^**	**1.56 × 10^−5^**
Unknown 89	105	991	1.01 × 10^−4^	7.75 × 10^−5^	7.67 × 10^−5^	
Unknown 90	81	991	1.12 × 10^−4^	8.46 × 10^−5^	4.52 × 10^−5^	0.000118
Unknown 92	29	1002	1.72 × 10^−3^	2.57 × 10^−3^	2.16 × 10^−3^	
Unknown 95	81	1025	4.55 × 10^−4^	1.39 × 10^−4^	1.09 × 10^−4^	0.000253
Unknown 100	71	1060	1.39 × 10^−4^	2.11 × 10^−4^	1.88 × 10^−4^	0.018067
**Unknown 102**	**87**	**1061**	**9.85 × 10^−6^**	**3.01 × 10^−4^**	**7.66 × 10^−4^**	**7.03 × 10^−8^**
Unknown 103	105	1067	1.88 × 10^−4^	7.88 × 10^−5^	9.52 × 10^−5^	7.16 × 10^−6^
Unknown 105	60	1077	1.42 × 10^−4^	1.29 × 10^−4^	7.21 × 10^−5^	
**Unknown 1117**	**267**	**1140**	**2.57 × 10^−3^**	**1.10 × 10^−3^**	**1.30 × 10^−3^**	**3.10 × 10^−7^**
Unknown 118	77	1171	1.45 × 10^−3^	5.58 × 10^−4^	2.42 × 10^−4^	0.004415
Unknown 120	60	1180	2.75 × 10^−3^	1.73 × 10^−3^	1.34 × 10^−3^	0.002391
**Unknown 121**	**104**	**1183**	**2.35 × 10^−5^**	**3.81 × 10^−5^**	**9.05 × 10^−5^**	**1.38 × 10^−6^**
**Unknown 125**	**85**	**1200**	**1.00 × 10^−4^**	**1.49 × 10^−4^**	**2.48 × 10^−4^**	**0.000259**
Unknown 1268	57	2479	1.00 × 10^−5^	4.20 × 10^−4^	1.00 × 10^−5^	1.22 × 10^−9^
Unknown 147	73	1313	7.80 × 10^−6^	3.17 × 10^−5^	4.48 × 10^−5^	5.67 × 10^−6^
Unknown 149	74	1326	8.87 × 10^−5^	6.77 × 10^−5^	6.43 × 10^−5^	0.033612
Unknown 150	71	1334	4.18 × 10^−5^	5.75 × 10^−5^	5.16 × 10^−5^	0.010128
**Unknown 151**	**121**	**1353**	**1.50 × 10^−5^**	**1.43 × 10^−5^**	**4.46 × 10^−5^**	3.26 × 10^−5^
**Unknown 166**	**70**	**1448**	**5.20 × 10^−5^**	**5.85 × 10^−5^**	**7.87 × 10^−5^**	**0.011226**
Unknown 169	85	1474	1.68 × 10^−4^	1.03 × 10^−4^	7.28 × 10^−5^	3.80 × 10^−6^
Unknown 171	88	1493	3.58 × 10^−4^	2.37 × 10^−4^	2.83 × 10^−4^	0.001454
Unknown 175	57	1506	1.05 × 10^−4^	7.77 × 10^−5^	7.86 × 10^−4^	
Unknown 178	74	1523	1.56 × 10^−4^	1.28 × 10^−4^	8.06 × 10^−5^	0.000858
**Unknown 185**	**88**	**1593**	**1.10 × 10^−2^**	**6.67 × 10^−3^**	**1.01 × 10^−2^**	**1.21 × 10^−5^**
Unknown 257	57	2340	2.96 × 10^−4^	6.40 × 10^−4^	1.66 × 10^−4^	0.002612
Unknown 259	57	2405	2.93 × 10^−4^	4.77 × 10^−4^	1.67 × 10^−4^	0.009889
Unknown 260	145	2437	4.70 × 10^−6^	1.31 × 10^−4^	2.16 × 10^−6^	0.002230
**Unknown 268**	**57**	**2495**	**2.84 × 10^−4^**	**1.00 × 10^−5^**	**1.38 × 10^−4^**	**7.51 × 10^−7^**
Unknown 276	99	2530	4.09 × 10^−5^	3.64 × 10^−4^	1.66 × 10^−5^	7.87 × 10^−7^
Unknown 285	127	1121	3.98 × 10^−7^	6.52 × 10^−5^	1.29 × 10^−6^	1.96 × 10^−5^
**Unknown 2001**	**71**	**1200**	**1.87 × 10^−4^**	**2.60 × 10^−4^**	**4.28 × 10^−4^**	**6.66 × 10^−5^**
**Unknown 2002**	**79**	**1037**	**2.45 × 10^−4^**	**1.16 × 10^−4^**	**3.41 × 10^−4^**	**1.17 × 10^−6^**

Mean values represent the mean TIC-corrected mean abundance for each feature. The 95% CI of the percentage relative standard deviation (%RSD) is to help assess variability.; *p*-value: significance of one-way ANOVA test; Bolded rows: features that are significantly enriched in the Cabernet-soaked cheese compared to the Alicante-soaked cheese.

**Table 6:** Sensory parameters scores for cheeses soaked in red wines and the control.

Sensory Parameter	Control	Cabernet Sauvignon	Alicante Bouchet
Flavor intensity	5.51 ± 1.96 ^a^	6.22 ± 1.89 ^b^	7.06 ± 1.49 ^b^
Aroma intensity	6.44 ± 1.48 ^a^	6.92 ± 1.87 ^b^	7.52 ± 1.30 ^b^
Texture	6.64 ± 1.65 ^a^	6.44 ± 1.79 ^a^	7.05 ± 1.49 ^a^
Rind’s color	6.23 ± 1.73 ^a^	7.15 ± 1.75 ^b^	7.14 ± 1.67 ^b^
Overall liking	5.43 ± 1.94 ^a^	6.40 ± 1.85 ^b^	7.25 ± 1.50 ^b^

Mean values ± s.d.; ^a,b^: values with different superscript per row were significantly different (*p* < 0.05).

## Data Availability

The original contributions presented in the study are included in the article, further inquiries can be directed to the corresponding author.

## References

[1] Biango-daniels M.N. (2021). American Artisan Cheese Quality and Spoilage: A Survey of Cheesemakers’ Concerns and Needs. J. Dairy Sci..

[2] Becker K.M., Parsons R.L., Kolodinsky J., Matiru G.N. (2007). A Cost and Returns Evaluation of Alternative Dairy Products to Determine Capital Investment and Operational Feasibility of a Small-Scale Dairy Processing Facility. J. Dairy Sci..

[3] Dudić B., Mittelman A., Gubíniová K., Pajtinková Bartáková G., Kader S., Zejak D., Spalević V. (2024). Wine Industry and Wine Markets: Dynamics, Challenges and Implications of Globalization. AGROFOR Int. J..

[4] Galmarini M.V., Dufau L., Loiseau A.L., Visalli M., Schlich P. (2018). Wine and Cheese: Two Products or One Association? A New Method for Assessing Wine-Cheese Pairing. Beverages.

[5] “Guffanti Formaggi” Formaggio Ubriacato. https://guffantiformaggi.com/en/cheese/formaggio-ubriacato/.

[6] López M.B., Ferrandini E., Rodriguez M., Roca J.D., Haba E., Luna A., Rovira S. (2012). Physicochemical Study of Murcia al Vino Cheese. Small Rumin. Res..

[7] Di Cagno R., Buchin S., De Candia S., De Angelis M., Fox P.F., Gobbetti M. (2007). Characterization of Italian Cheeses Ripened under Nonconventional Conditions. J. Dairy Sci..

[8] Gyenge L., Erdő K., Albert C., Laslo É., Salamon R.V. (2024). The Effects of Soaking in Salted Blackcurrant Wine on the Properties of Cheese. Heliyon.

[9] Innocente N., Biasutti M., Comuzzo P. (2007). Characterization of a Traditional Semi-Hard Italian Cheese Produced by Soaking in Wine. Food Chem..

[10] Ferrandini E., Castillo M., de Renobales M., Virto M.D., Garrido M.D., Rovira S., López M.B. (2012). Influence of Lamb Rennet Paste on the Lipolytic and Sensory Profile of Murcia al Vino Cheese. J. Dairy Sci..

[11] Bertuzzi A.S., McSweeney P.L.H., Rea M.C., Kilcawley K.N. (2018). Detection of Volatile Compounds of Cheese and Their Contribution to the Flavor Profile of Surface-ripened Cheese. Compr. Rev. Food Sci. Food Saf..

[12] Andiç S., Tunçtürk Y., Boran G., Preedy V. (2014). Chapter 28. Changes in Volatile Compounds of Cheese. Processing and Impact on Active Components in Food.

[13] Rizzo P.V., Del Toro-Gipson R.S., Cadwallader D.C., Drake M.A. (2022). Identification of Aroma-Active Compounds in Cheddar Cheese Imparted by Wood Smoke. J. Dairy Sci..

[14] Faulkner H., O’Callaghan T.F., McAuliffe S., Hennessy D., Stanton C., O’Sullivan M.G., Kerry J.P., Kilcawley K.N. (2018). Effect of Different Forage Types on the Volatile and Sensory Properties of Bovine Milk. J. Dairy Sci..

[15] Garofalo G., Busetta G., Alfonzo A., Francesca N., Moschetti G., Settanni L., Gaglio R. (2022). Effetto Dell’immersione in Vino Rosso Sul Profilo Microbiologico, Contenuto Totale in Composti Fenolici e Aspetti Sensoriali Di Un Formaggio Ovino a Pasta Pressata. Sci. E Tec. Latt. Casearia.

[16] Tejada L., Abellán A., Cayuela J.M., Martínez-Cacha A. (2006). Sensorial Characteristics during Ripening of the Murcia al Vino Goat’s Milk Cheese: The Effect of the Type of Coagulant Used and the Size of the Cheese. J. Sens. Stud..

[17] AOAC (2000). AOAC Official Method 989.10. Coliform and Escherichia Count in Food. Dry Rehydratable Film Method (PetrifilmTM Method). Official Methods of Analysis of AOAC International.

[18] AOAC (2002). AOAC Official Method 996.02. Coliform Count in Dairy Products. Official Methods of Analysis of AOAC International.

[19] Licón C.C., Carmona M., Molina A., Berruga M.I. (2012). Chemical, Microbiological, Textural, Color, and Sensory Characteristics of Pressed Ewe Milk Cheeses with Saffron *(Crocus sativus* L.) during Ripening. J. Dairy Sci..

[20] Pérez-Magariño S., González-Sanjosé M.L. (2003). Application of Absorbance Values Used in Wineries for Estimating CIELAB Parameters in Red Wines. Food Chem..

[21] ColorDesigner LAB to RGB Color Converter. https://colordesigner.io/convert/labtorgb.

[22] Osunbade O.A., Ajiboye T.S., Adisa O.A., Olatunji O., Oyewo I. (2021). Evaluation of Consumers Acceptability of Cassava Cooked Paste (EBA) Using 9-Points Hedonic, Jar, and Cata Methods. Int. J. Multidiscip. Res. Publ. (IJMRAP).

[23] Rahayu N.I., Muktiarni M., Hidayat Y. (2024). An Application of Statistical Testing: A Guide to Basic Parametric Statistics in Educational Research Using SPSS. ASEAN J. Sci. Eng..

[24] Guler Z., Park Y., Sekerli Y. (2014). Evaluation of Volatile Compounds of Red and White Wine Treated Dil Cheese during Refrigerated Storage. Front. Food Sci. Technol..

[25] Gaglio R., Barbaccia P., Barbera M., Restivo I., Attanzio A., Maniaci G., Di Grigoli A., Francesca N., Tesoriere L., Bonanno A. (2021). The Use of Winery By-Products to Enhance the Functional Aspects of the Fresh Ovine “Primosale” Cheese. Foods.

[26] Tunick M.H. (2022). Aging of Hispanic Cheese. ACS Symp. Ser..

[27] Giroux H.J., Lemaire N., Britten M. (2022). Effect of Cheese Composition and Cheese-Making Conditions on Salt and Moisture Transfer during Brining. Int. Dairy J..

[28] Fox P.F., Guinee T.P., Cogan T.M., McSweeney P.L.H. (2017). Fundamentals of Cheese Science.

[29] Guinee T.P., Fox P.F. (2017). Salt in Cheese: Physical, Chemical and Biological Aspects. Cheese: Chemistry, Physics and Microbiology.

[30] Revilla E., Losada M.M., Gutiérrez E. (2016). Phenolic Composition and Color of Single Cultivar Young Red Wines Made with Mencia and Alicante-Bouschet Grapes in AOC Valdeorras (Galicia, NW Spain). Beverages.

[31] Wen P., Zhu Y., Luo J., Wang P., Liu B., Du Y., Jiao Y., Hu Y., Chen C., Ren F. (2021). Effect of Anthocyanin-Absorbed Whey Protein Microgels on Physicochemical and Textural Properties of Reduced-Fat Cheddar Cheese. J. Dairy Sci..

[32] Vaquero M.J.R., Alberto M.R., de Nadra M.C.M. (2007). Antibacterial Effect of Phenolic Compounds from Different Wines. Food Control.

[33] Cabezas L., Sanchez I., Poveda J.M., Seseña S., Palop M. (2005). Comparison of Microflora, Chemical and Sensory Characteristics of Artisanal Manchego Cheeses from Two Dairies. Food Control.

[34] Poveda J.M., Chicón R., Cabezas L. (2015). Biogenic Amine Content and Proteolysis in Manchego Cheese Manufactured with Lactobacillus Paracasei Subsp. Paracasei as Adjunct and Other Autochthonous Strains as Starters. Int. Dairy J..

[35] USDA Food Safety and Inspection Service Microbiological Testing by Industry of Ready-to-Eat Foods Under FDA’s Jurisdiction for Pathogens (or Appropriate Indicator Organisms): Verification of Preventive Controls. https://www.fsis.usda.gov/sites/default/files/media_file/2022-03/NACMCF_2018-2020_RTE_Testing.pdf.

[36] Sabel A., Bredefeld S., Schlander M., Claus H. (2017). Wine Phenolic Compounds: Antimicrobial Properties against Yeasts, Lactic Acid and Acetic Acid Bacteria. Beverages.

[37] Torres-Guardado R., Esteve-Zarzoso B., Reguant C., Bordons A. (2022). Microbial Interactions in Alcoholic Beverages. Int. Microbiol..

[38] Marín P., Palmero D., Jurado M. (2015). Occurrence of Moulds Associated with Ovine Raw Milk and Cheeses of the Spanish Region of Castilla La Mancha. Int. J. Dairy Technol..

[39] Jurado M., Vicente C.J. (2020). Penicillium Commune Affects Textural Properties and Water Distribution of Hard and Extra-Hard Cheeses. J. Dairy Res..

[40] Schymanski E.L., Jeon J., Gulde R., Fenner K., Ruff M., Singer H.P., Hollender J. (2014). Identifying small molecules via high resolution mass spectrometry: Communicating confidence. Environ. Sci. Technol..

[41] Gürbüz O., Rouseff J.M., Rouseff R.L. (2006). Comparison of Aroma Volatiles in Commercial Merlot and Cabernet Sauvignon Wines Using Gas Chromatography− Olfactometry and Gas Chromatography− Mass Spectrometry. J. Agric. Food Chem..

[42] de Macêdo Morais S., de Sousa Galvão M., Olegario L.S., de Carvalho L.M., Pereira G.E., de Andrade Lima L.L., da Silva F.L.H., Madruga M.S. (2022). Identification of Chemical Markers of Commercial Tropical Red Wine Candidates for the São Francisco Valley Geographical Indication. Food Anal. Methods.

[43] Le Quéré J.-L., Pierre A., Riaublanc A., Demaizières D. (1998). Characterization of Aroma Compounds in the Volatile Fraction of Soft Goat Cheese during Ripening. Le Lait.

[44] Curioni P.M.G., Bosset J.O. (2002). Key Odorants in Various Cheese Types as Determined by Gas Chromatography-Olfactometry. Int. Dairy J..

[45] Carunchia Whetstine M.E., Cadwallader K.R., Drake M. (2005). Characterization of Aroma Compounds Responsible for the Rosy/Floral Flavor in Cheddar Cheese. J. Agric. Food Chem..

[46] Waterhouse A.L., Sacks G.L., Jeffery D.W. (2024). Understanding Wine Chemistry.

[47] Roger S., Degas C., Gripon J.C. (1988). Production of Phenyl Ethyl Alcohol and Its Esters during Ripening of Traditional Camembert. Food Chem..

[48] Xiao Q., Zhou X., Xiao Z., Niu Y. (2017). Characterization of the Differences in the Aroma of Cherry Wines from Different Price Segments Using Gas Chromatography–Mass Spectrometry, Odor Activity Values, Sensory Analysis, and Aroma Reconstitution. Food Sci. Biotechnol..

[49] Li S., Li Y., Du Z., Li B., Liu Y., Gao Y., Zhang Y., Zhang K., Wang Q., Lu S. (2021). Impact of NSLAB on Kazakh Cheese Flavor. Food Res. Int..

[50] Xu Y., Fan W., Qian M.C. (2007). Characterization of Aroma Compounds in Apple Cider Using Solvent-Assisted Flavor Evaporation and Headspace Solid-Phase Microextraction. J. Agric. Food Chem..

[51] Sgarbi E., Lazzi C., Tabanelli G., Gatti M., Neviani E., Gardini F. (2013). Nonstarter Lactic Acid Bacteria Volatilomes Produced Using Cheese Components. J. Dairy Sci..

[52] Culleré L., Ferreira V., Cacho J. (2011). Analysis, Occurrence and Potential Sensory Significance of Aliphatic Aldehydes in White Wines. Food Chem..

[53] Martín A., Carrillo F., Trillo L.M., Roselló A. (2009). A Quick Method for Obtaining Partition Factor of Congeners in Spirits. Eur. Food Res. Technol..

[54] Yu C., Zhu J., Chen J., Yu W., Wang C., Gu S., Huang D., Pu L., Qiu S., Dai Y. (2025). (*R*)-Ethyl Lactate Is an Important Contributor to Fruity Flavor of Baijiu as Revealed by Chiral GC–MS and Sensory Evaluation. Food Chem. X.

[55] Van Wyk C.J., Augustyn O.P.H., De Wet P., Joubert W.A. (1979). Isoamyl Acetate—A Key Fermentation Volatile of Wines of Vitis Vinifera Cv Pinotage. Am. J. Enol. Vitic..

[56] Chira K., Jourdes M., Teissedre P.-L. (2012). Cabernet Sauvignon Red Wine Astringency Quality Control by Tannin Characterization and Polymerization during Storage. Eur. Food Res. Technol..

[57] OIV (2017). Distribution of the World’s Grapevine Varieties.

[58] Fox P.F., McSweeney P.L.H. (2017). Cheese: An Overview. Cheese: Chemistry, Physics and Microbiology.

